# Quantifying relevant exposure determinants and conditions of use for welding emissions

**DOI:** 10.12688/openreseurope.19498.3

**Published:** 2025-06-13

**Authors:** Antti Joonas Koivisto, Maxime Eliat, Michael Jayjock, Tareq Hussein, Alessia Nicosia

**Affiliations:** 1 ARCHE Consulting, Wondelgem, B-9032, Belgium; 2 Institute for Atmospheric and Earth System Research (INAR/Physics), University of Helsinki, Helsinki, FI-00014, Finland; 3 Jayjock Associates, Langhorne, Pennsylvania, 19047, USA; 4 Environmental and Atmospheric Research Laboratory (EARL), The University of Jordan, Amman, Amman Governorate, 11942, Jordan; 5 CNR-ISAC, Institute of Atmospheric Sciences and Climate, National Research Council of Italy, Bologna, 40129, Italy

**Keywords:** Regulatory exposure assessment, Conditions of use, exposure modelling, risk assessment, emission, nanoparticle, inhalation exposure

## Abstract

**Background:**

Conditions of Use (CoU) defines the operational conditions and risk management measures that result in adequately controlled exposure. It is mandatory to provide CoUs in Exposure Scenarios for the REACH legislation. CoU assessment based on similar exposure groups is a case-specific approach and not very efficient. We demonstrate how to quantify relevant exposure determinants and find optimal CoUs for welding using probabilistic exposure modelling.

**Methods:**

Single and two-compartment models were applied to calculate exposure levels under specified conditions for five welding processes with the highest emissions. Model performance was tested using independently reported emissions and two field measurement studies.

**Results:**

Welding fume concentrations were predicted within the range of 0.93 to 3.4 times the measured levels for three different welding scenarios, where two scenarios were repeated with four and five different ventilation rates. CoUs were quantified for welding with five different electrodes when welding is performed without local exhaust ventilation controls, with a fume extraction torch, and with local exhaust ventilation. The exposure level was adequately controlled for three electrodes when controls were applied. Exposure assessment refinement proposals were given based on the relevant exposure determinants. The maximum emission factor was calculated for total fume emissions. A proposal for emission labelling was established.

**Conclusions:**

CoU quantification was successfully demonstrated for total welding fume emissions. CoU results can be applied for welding processes where emissions are less than specified for the operational conditions specified here. Only total fume emissions were considered here, which may not be the limiting factor for risk; volatile and individual metal emissions were not considered. The approach applies to point sources for which emissions are adequately characterized. CoU assessment can be used to develop emission labelling and process specific safety actions. Compared to the traditional case-specific approach, modeling using this general approach will significantly improve cost-effectiveness.

## Plain Language Summary

Conditions of Use (CoU) defines the operational conditions and risk management measures that result in adequately controlled exposure. In REACH legislation, the registrant must describe CoU for all contributing scenarios to ensure safe material use and process application. Here, we present how to quantify relevant exposure determinants and optimize CoU using process emissions and quantitative exposure modelling. Optimization is based on finding the least restrictive operational conditions under which the exposure is adequately controlled or minimizing personal exposure measurements and safety actions. This methodology was demonstrated for welding processes, but the methodology applies to all point sources.

## List of abbreviations

**Table:** 

ACH	Air Changes per Hour
AIHA	American Industrial Hygiene Association
CoU	Conditions of Use
DNEL	Derived-No-Effect-Level
ECHA	European Chemicals Agency
FET	Fume Extraction Torch
FF	Far-Field
GM	Geometric Mean
GMAW	Gas Metal Arc Welding
LEV	Local Exhaust Ventilation
NF	Near-Field
NOAEL	No-Observed-Adverse-Effect-Level
OEL	Occupational Exposure Limit
P90	Exposure distribution 90 ^th^ percentile
P95	Exposure distribution 95 ^th^ percentile
RCR	Risk Characterization Ratio
REACH	Registration, Evaluation, Authorisation, and Restriction of Chemicals
SMAW	Shielded Metal Arc Welding
TEAS	Task Exposure Assessment Simulator

## Introduction

Conditions of Use (CoU) defines the operational conditions and risk management measures that result in adequately controlled exposure. In REACH legislation, the registrant is required to provide arguments that adverse health effects are unlikely to occur under the CoU described for the exposure scenario ( ECHA, 2016). Quantitative exposure estimates are required for all contributing scenarios where hazardous emissions occur to support the risk characterization ( ECHA, 2016).

**Figure 1.**
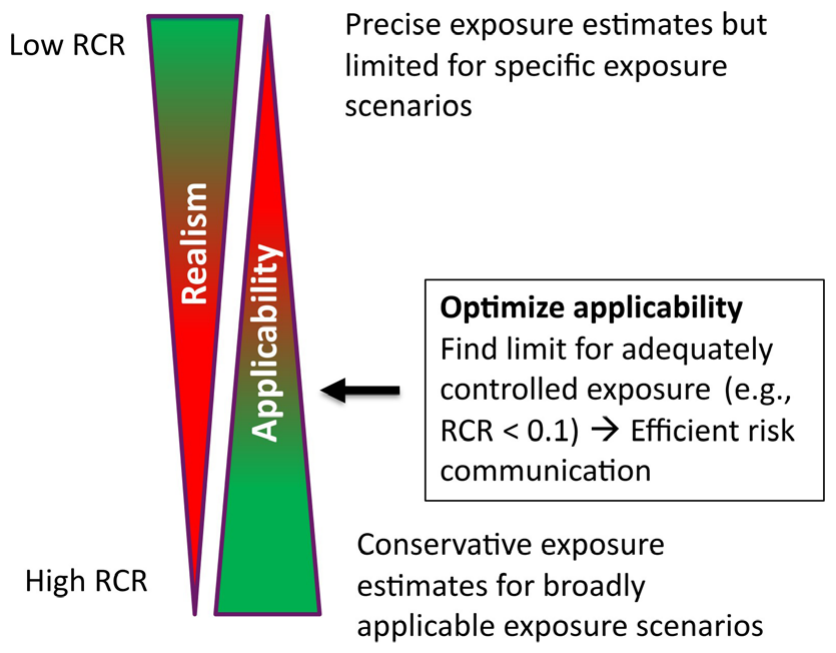
Optimizing CoU; RCR is risk characterization ratio defined as exposure/limit value.

In the framework of Directive 98/24/EC on chemical agents at work, worker exposure by inhalation is traditionally quantified by measuring personal breathing zone concentration across the work shift ( EN 689:2019+AC:2019). However, personal measurements can be applied only for the specific operational conditions under which the measurement was carried out, i.e., for similar exposure groups. The conditions for a similar exposure group require that all operational conditions, processes and materials, and working practices related to tasks and their durations are similar ( EN 689:2019). Personal measurement provides the actual exposition under specific conditions, but its use is limited in generic exposure assessment. A more feasible approach is to quantify the least restricting operational conditions under which the exposure is adequately controlled ( Figure 1; Koivisto *et al.*, 2021). This can be evaluated by using a quantitative exposure model where the exposure levels are below the exposure limit values.

The exposure limit is typically an occupational exposure limit (OEL) or similar health-based limit value (e.g., Derived-No-Effect-Level (DNEL) or No-Observed-Adverse-Effect-Level (NOAEL)) ( ECHA, 2016). In REACH legislation, the exposure is considered adequately controlled if the exposure distribution 90 ^th^ percentile (P90) is below the limit value, i.e., P90<OEL ( ECHA, 2016). However, this allows 10% of the workers to experience overexposure, which generally requires safety control actions.

A more convenient and transparent approach is the American Industrial Hygiene Association (AIHA) exposure category scheme that classifies exposure based on the exposure distribution 95 ^th^ percentile (P95) as: 1. *non-existent* (P95 ≤ 0.01×OEL), 2. *highly controlled* (0.01×OEL ≤ P95 ≤ 0.1×OEL), 3. *well controlled* (0.1×OEL ≤ P95 ≤ 0.5×OEL), 4. *controlled* (0.5×OEL ≤ P95 ≤ OEL), and 5. *poorly controlled* (P95 > OEL) ( Hewett *et al.*, 2006; Torres *et al.*, 2014). Based on the exposure rating, a list of actions needs to be conducted to minimize the overexposure potential ( Hewett *et al.*, 2006).

CoU assessment can be used to justify the minimum requirement for personal exposure measurements. According to EN 689:2019, the personal exposure measurement interval is 36 months (most extended interval) if the geometric mean (GM) exposure level is <0.1×OEL.

Based on the CoU assessment objectives, the following limits can be considered:

- P90 < OEL or DNEL or NOAEL for REACH compliance ( ECHA, 2016),
- GM < 0.1×OEL for minimizing personal exposure measurements ( EN 689:2019), and
- P95 ≤ 0.1×OEL ( *highly controlled*) to manage the risk by “ *General or chemical specific hazard communication*” ( Hewett *et al.*, 2006).

The exposure limit is recommended to be set as P95 ≤ 0.1×OEL, corresponding to the “ *highly controlled*” AIHA exposure category to minimize the overexposure risk and risk management actions and to minimize personal exposure measurements. Allocation factors may also be considered if there are multiple sources for the specific contaminant in the same space or if integrated exposure needs to be considered (public exposure).

Process emissions are scarcely reported, especially for particulate matter ( Abadie, 2021; Abadie & Blondeau, 2011; Koivisto *et al.*, 2017; US EPA, 2016). This is exceptional considering risk assessment because emissions are the primary drivers of exposure and health effects. Emission characterization is also necessary to assess industrial process machines’ emissions and risks and mitigate them by design ( EN 1093-1:2008; EN ISO 12100: 2010; EN ISO 14123-1:2015). We hypothesize that emissions are not systematically reported because 1) ECHA requires information only on exposure potential that can be calculated using models that do not require quantitative information about emissions, and 2) personal exposure measurements are still considered a more feasible approach to assess compliance than exposure modelling. However, such assessment is not generic, and repeated assessments are needed. A more convenient and efficient approach is quantifying process emissions and CoU for a specific process.

This study demonstrates how to quantify relevant exposure determinants and CoU for welding. Welding emissions were selected for demonstration because the emissions are reasonably well-studied ( Quecke *et al.*, 2023) and they pose relevant risks unless proper emission controls are applied ( Kendzia *et al.*, 2019). There also exists exposure studies with sufficient contextual information for model performance testing ( Boelter *et al.*, 2009; Harris *et al.*, 2005). Exposure assessment was performed by using a near-field/far-field model that has been shown to predict worker exposure to welding fumes well (R ^2^ = 0.81–0.94) ( Wang *et al.*, 2022) and has been applied for welding fume exposure assessment ( Lopes *et al.*, 2024). First, we demonstrate how relevant exposure determinants can be evaluated for a single-point source. Then, we demonstrate how well independently characterized welding emissions can be used to assess welder exposure under realistic welding conditions using mechanistic exposure models. Finally, we assess CoUs for specific welding processes.

## Methods

### Welding emissions

Gas metal arc welding (GMAW) was selected to demonstrate how to quantify process-specific CoU because welding emissions are reasonably well-studied (e.g., Quecke *et al.*, 2023; US-EPA, 1994). Relevant emission determinants are electrode type, electrode diameter, current, voltage, and shielding gas composition ( Jilla, 2019; Satheesh Kumar *et al.*, 2021). The emission factor (mg/g-wire) for welding fumes is less affected by the wire feed rate, shielding gas flow rate, contact tip to work distance, and base metal ( Jilla, 2019; Popović *et al.*, 2014). Welding power (product of current and voltage) generally increases emissions but not linearly ( Satheesh Kumar *et al.*, 2021; Yamazaki *et al.*, 2012). More than 90% of the emissions are associated with the electrode ( HSL, 2000; Jilla, 2019), and the emission factor generally increases when electrode diameter decreases (note, wire use rate decreases as well unless wire speed increases) ( Jilla, 2019).

Welding emissions were extracted from Quecke *et al.* (2023) and references therein. Relevant emission determinants were extracted and reported for the highest emissions.

### Exposure modelling

Exposure modelling was conducted using Task Exposure Assessment Simulator (TEAS), version 1.05 (2019), Exposure Assessment Solutions, Inc., Missouri, U.S., www.easinc.co (Professional license). TEAS well-mixed air model (1Box.CE.Gv; Hewett & Ganser, 2017) and a Near-Field/Far-Field (NF/FF) model without local exhaust ventilation (LEV) (2Box.CE.Gv; Ganser & Hewett, 2017) and with local exhaust ventilation where the air is extracted to the outdoors (2Box.CE.Lev.Gv; Ganser & Hewett, 2017) were used in this study. TEAS is a similar exposure model to the free software IH-MOD 2.0 provided by the AIHA ( https://ihmod.org/).

A well-mixed air model assumes that air in the room is thoroughly mixed at all times and there are no other losses for concentrations than general ventilation exhaust. Such a model was extensively evaluated for its sensitivity against penetration, air exchange (i.e. ventilation), dry deposition, and emissions by Hussein *et al.* (2021) for the purpose of exposure assessment.

A NF/FF model describes the concentrations near the source (near-field; NF) and further away from the source (far-field; FF). In the NF/FF model, a room is divided into two volumes where NF volume ( *V _NF_*, m ^3^) compromises the source ( *S _m_*, mg/min) and possibly a worker breathing zone and the FF volume ( *V _FF_*, m ^3^) rest of the room ( *i.e. V _room_* = *V _NF_* + *V _FF_*). The room is ventilated via FF volume ( *Q*, m ^3^/min), and the incoming ventilation air concentration is assumed to be zero. The room air exchange is calculated by using the total room volume. There is a limited air exchange between the NF and FF volumes ( *β*, m ^3^/min). The NF/FF model, in its general form, assumes that:

1) All mass entering the model volume is created at a source inside the NF volume or from incoming ventilation air,
2) Particles are always thoroughly mixed in the NF and FF volumes,
3) There is limited air exchange between NF and FF volumes, and
4) There are no other particle losses than from the FF ventilation.

A NF/FF model with LEV assumes that ventilation air is extracted from the NF volume and exhausted outdoors. LEV air flow increases the NF-FF flow rate and the general ventilation incoming air volume flow to the FF. General ventilation is not assumed to be affected by the LEV flow rate, *i.e.*, general ventilation air changes per hour (ACH) is calculated from general ventilation exhaust flow, independent of the LEV flow rate.

## Results and discussion

### Quantifying relevant exposure determinants

The exposure determinant is considered relevant when it significantly impacts exposure level. Exposure determinant relevance can be quantified using a probabilistic exposure model ( Koivisto *et al.*, 2021). Here, a probabilistic NF/FF model 2Box.CE.Gv ( Ganser & Hewett, 2017) was used to assess the effect of general ventilation, room volume, near-field volume, and emission rate on the concentration level. Each parameter range was changed by one order of magnitude (uniform distribution). Work shift duration was set to 480 min, the process was continuous, and all emissions were assumed to occur in the NF (point source). Simulation reports for NF and FF concentration calculations are available in Extended data, Section 1; Koivisto *et al.*, 2025).

**Figure 2.**
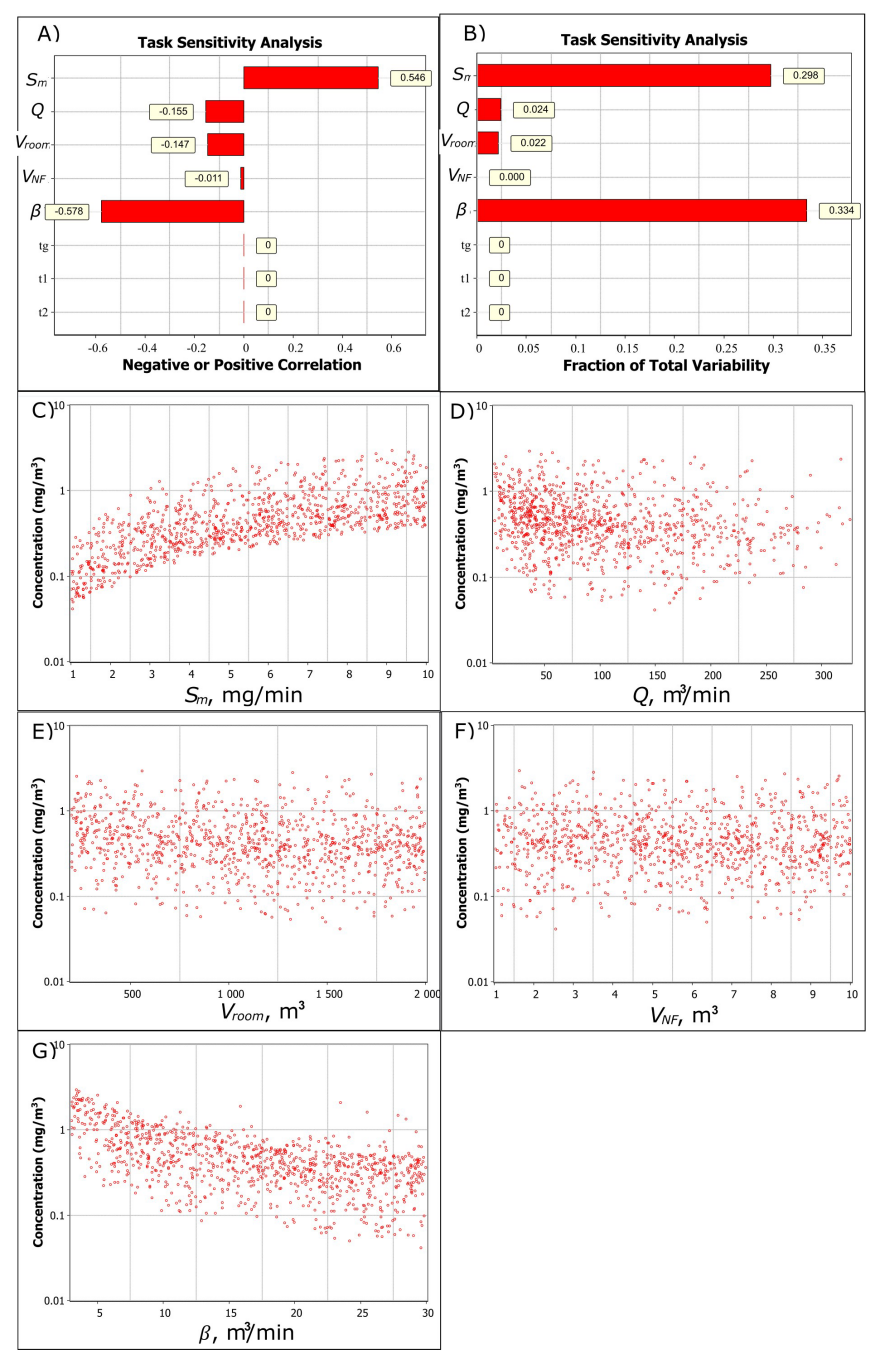
Task sensitivity analysis for the NF total fume concentration. **A**) Spearman correlation coefficients showing if the exposure determinant effect on exposure is positive or negative by increasing parameter value and **B**) total variability showing how the exposure determinants cause the variation in the simulated NF concentration level, and the relation of **C**) emission rate, **D**) ventilation volume flow, **E**) room volume, **F**) near-field volume, and **G**) NF-FF air exchange to the NF concentration. In **A**) and **B**) generation time ( *t _g_*), worker start- ( *t _1_*) and end ( *t _2_*) times in the NF are constant values.

Sensitivity analysis shows that for NF concentration, there is a strong positive correlation (0.546) related to increasing emission rate and a strong negative correlation (-0.578) related to increasing NF-FF air exchange ( Figure 2A). Increasing general ventilation rate and room volume have a less significant negative correlation of -0.155 and -0.147, respectively, to the NF concentration ( Figure 2B). The highest variability related to the NF concentration is caused by emission rate and the NF-FF air exchange and, to a lesser extent, general ventilation, room volume, and NF volume ( Figure 2B). Figures 2C to 2G show the trend in increasing parameter values (note, log y-axis). It shows that the NF concentration increases linearly with the emission rate, general ventilation reduces concentrations at below 50 m ^3^/min ventilation flows, room volume or NF volume does not have a significant effect on the concentration, and NF-FF air exchange effect on NF concentration reduces (approaches a to thoroughly mixed air). For example, the geometric mean concentration for *V _NF_* = 1 m ^3^ is 0.44 mg/m ^3^, and for *V _NF_* = 10 m ^3^ is 0.43 mg/m ^3^. This is explained by the rapid saturation of concentration in the NF volume regardless of whether the volume is 1 or 10 m ^3^.

Respectively, for FF concentration level there is a strong positive correlation (0.377) for emission rate and a strong negative correlation for general ventilation and room volume (-0.526 and -0.476, respectively). NF volume and NF-FF air exchange are not significant factors for FF concentration.

This shows that emission rate and NF-FF air exchange should be accurately characterized for the NF exposure to provide good model predictability. For the FF exposure, relevant exposure determinants are emission rate, general ventilation flow rate, and room volume.

### Welding exposure model performance testing

Here, the model performance testing was conducted using independently evaluated emission rates, which were then applied to a realistic exposure scenario to calculate exposure levels. This allows for the evaluation of emission measurements performed in a chamber as to whether they are affected by working practices or are influenced by different environmental conditions, such as wind speed, relative humidity, base material, or temperature.

Performance testing was conducted by using Harris *et al.* (2005) scenarios with different ventilation rates and Boelter *et al.* (2009) “boiler room” welding scenario (another scenario did not have sufficient contextual information). Welding was performed by using shielded metal arc welding (SMAW). All welding parameters were not reported by Harris *et al.* (2005) and Boelter *et al.* (2009) and thus, emissions were extrapolated according to our best understanding from Quecke *et al.* (2023). It is reported that relevant emission factors for shielded metal arc welding (SMAW) are electrode type, diameter, use rate, voltage, current, and coating ( Quecke *et al.*, 2023). In Harris *et al.* (2005), the room air was well mixed based on the upstream and downstream concentration measurements. Boelter *et al.* (2009) calculated the NF-FF air exchange based on the NF and FF concentration measurements that resulted in an average of 21 m ^3^/h. Welding parameters and operational conditions applied in model performance testing are given in Table 1.

**Table 1.** Operational conditions in welding scenarios for model performance testing.

Parameter	( Harris *et al.*, 2005)		( Boelter *et al.*, 2009)
Welding type	SMAW		SMAW
Electrode	E6010	E7018	E6010 and E7018
Electrode manufacturer	The Lincoln Electric Company		The Lincoln Electric Company
Electrode model	N/A		E6010 Fleetweld 5P and E7018 Jetweld LH-70 H4R
Electrode diameter	3.2 mm		E6010: 3.2 mm E7018: 2.4 mm
Current	105 A	125 A	70 to 150 A
Voltage	N/A		7 to 9 V
Electrode use rate	12.9 g/min	18.2 g/min	12.9 g/min assumed
Arc time	N/A (continuous is assumed)		56 min (Electrode-specific arc time was not reported)
Base material	Mild carbon steel		Carbon steel
Emission rates	Sowards *et al.* (2008): 387 mg/min (93 A, 27.1 V, electrode diameter N/A) to 598 mg/min (115 A, 30.8 V), uniform distribution US-EPA (1994): 25.6 mg/g-electrode, 329 mg/min for 12.9 g/min use rate.	Keane et al. (2014): 320 mg/min (125 to 150 A, 20 V, electrode diameter 4.8 mm) ( Sham & Liu, 2013): 180 and 310 mg/min (electrode diameters 3.2 mm and 4 mm, respectively) Sowards *et al.* (2008): 365 mg/min (126 A, 23.8 V, electrode diameter 3.2 mm) The emission rate was selected as 180 to 365 mg/min, a uniform range.	Emission rates for E6010 electrode was 387 to 589 mg/min, uniform range, and E7018 electrode was 180 to 365 mg/min, uniform range, and Electrode arc time was selected as 50%/50% for E6010/E7018.
Room volume	62.1 m ^3^		81 m ^3^
ACH	60.4, 44.6, 28.4, 14.4, and 6.6 1/h		23 1/h
Beta	N/A (thoroughly mixed based on upstream and downstream measurements)		21 m ^3^/min (average)
Sampling duration	60		325

Concentrations in Harris *et al.* (2005) scenarios were calculated using a well-mixed air model (1Box.CE.Gv; Hewett & Ganser, 2017) and in Boelter *et al.* (2009) the scenario was calculated using a NF/FF model (2Box.CE.Gv; Ganser & Hewett, 2017). Modelling reports are available in Extended data, Sections 2 to 5 ( Koivisto *et al.*, 2025).

For Harris *et al.* (2005) E6010 welding scenario, the model predictability ranged from 1.6 to 3.4 for emissions reported by Sowards *et al.* (2008) and from 1.0 to 2.3 for emissions calculated by using US-EPA (1994) emissions ( Table 2). For Harris *et al.* (2005) E7018 welding scenario, the model predictability ranged from 0.93 to 2.0 when using an aggregated emission rate ( Keane *et al.*, 2014; Sham & Liu, 2013; Sowards *et al.*, 2008). For low ventilation rates, the modelled concentration levels were more overestimated than for higher ventilation rates ( Table 2). For Boelter *et al.* (2009), the modelled geometric mean and measured concentration ratios were 0.99 and 1.53 for NF and FF, respectively. The NF/FF model performance is in line with the NF/FF model performance for volatiles ( Abattan *et al.*, 2021) and welding fumes ( Wang *et al.*, 2022).

**Table 2.** Modelled/measured concentration ratios for Harris *et al.* (2005) welding scenarios.

ACH, 1/h	E6010, Sowards *et al.* (2008): 387 to 589 mg/min, uniform distribution, GM	E6010, US-EPA (1994): 329 mg/min	E7018, aggregated emissions from Keane *et al.* (2014), Sham & Liu (2013), and Sowards *et al.* (2008): 180 to 365 mg/min, uniform range.
60.4	1.6	1.1	0.93
44.6	1.5	1.0	0.93
28.4	No data	No data	1.2
14.4	2.4	1.6	1.5
6.6	3.4	2.3	2.0

Unknown uncertainties are mainly related to electrode voltage that was not reported by Harris *et al.* (2005) and in Boelter *et al.* (2009) the voltage was significantly lower than used in Keane *et al.* (2014) and Sowards *et al.* (2008) ( Sham & Liu (2013) did not report voltage) ( Table 2). However, the model comparison with measurements shows that the predictability is in the range as demonstrated by ( Jayjock *et al.*, 2011) and Abattan *et al.* (2021) for NF/FF model for volatile emissions. This can be expected because particles emitted by welding are mainly below 1 µm (in mass concentration; Sowards *et al.*, 2008) for which deposition via gravitational settling is not a relevant sink. This makes the emission loss rates comparable with low- or non-reactive volatile emissions.

### Quantifying CoU for Gas Metal Arc Welding (GMAW)

CoU assessment is demonstrated for the manual GMAW process performed 1) without controls, 2) using a fume extraction torch (FET), and 3) using a fixed/mobile LEV. Other controls, such as downdraft bench or welding chamber, were not considered here.

***Exposure limit value***

CoU assessment starts by identifying relevant emission components, including volatiles, that cause the highest risk potential based on their emissions and exposure limit values. For simplicity, total fume emission is assumed to be the restricting component. The majority of the countries have a 5 mg/m ^3^ limit value as 8-h time weighted average for welding fumes, and the lowest value is 1 mg/m ^3^ (Netherlands) ( IARC, 2017).

***Welding emissions***

The CoUs were calculated for different GMAW electrodes whose maximum emissions are given in Table 3, as reported by ( Quecke *et al.*, 2023). The GMAW welding process particle emissions are mainly submicrometric with mass mean aerodynamic diameter ranging from 0.19 to 0.41 µm and geometric standard deviation from 1.3 to 1.8, depending on the welding parameters and shielding gas ( Cena *et al.*, 2014; Keane *et al.*, 2009). Wire feed rate and arc time specify the total amount of wire used during welding and the total emissions, which are calculated by multiplying the emission factor (mg/g-wire) by the wire use amount/rate (g-wire/min). The welding machine can control the wire feed rate. Here, the CoU was calculated for wire density of 7.8 g/cm ^3^ and wire feed rate ranging from 50 to 250 mm/s (uniform distribution, average 150 mm/s) ( Table 3).

**Table 3.** Maximum emissions measured for different GMAW welding process parameters.

Parameter	( Satheesh Kumar *et al.*, 2021)	( Jilla, 2019)	( Jilla, 2019)	Keane *et al.* (2014)	( Yamazaki *et al.*, 2012)
Wire type	ER316 L	ER316LSi	ER70S-6	ER70S-3	Z 3312 YGW11
Wire diameter, mm	1.2	1.2	1.2	1.14	1.2
Current, A	200	240	180	230-260	280-290
Voltage	N/A	26	26	24-26	31
Wire feed rate, mm/s	3.3	163	85	127	200
Maximum emission factor reported by Quecke *et al.* (2023)	22.1 mg/g for CO _2_ 5 LPM	5.59 mg/g for 99% Ar + 1% O _2_ 16.5 LPM	10.21 mg/g for 90% Ar + 10% CO _2_ 16.5 LPM	6.4±0.5 mg/g for 90% Ar + 10% CO _2_ 19 LPM	6.0 mg/g for CO _2_ 25 LPM
Wire mass feed rate, g/s	0.44 to 2.20	0.44 to 2.20	0.44 to 2.20	0.40 to 1.99	0.44 to 2.20
*S _m_* range (average). mg/min	584 to 2921 (1753)	148 to 739 (444)	270 to 1350 (810)	153 to 764 (458)	159 to 794 (159)

***Arc time***

Emissions occur when the arc is active. Typical arc time in a semi-automatic welding operation ranges on average from 10% to 12% ( Miller, 2020). Boelter *et al.* (2009) measured arc time up to 25% during 107 min task and 28% arc time during 15-min work period. Here, the arc time was selected to range from 12% to 20% (uniform distribution) during an 8-h work shift corresponding to 58 to 96 min arc time. The lower limit (58 min) represents an active welding day, and the upper limit (96 min) represents an intensive welding day. In electrode consumption, this corresponds to 4.6 to 7.6 kg wire consumption per workday (diameter 1.2 mm; average feed rate 150 mm/s). This is considered a reasonable maximum arc time range for manual welding.

***Emission controls for GMAW***

The FET capturing efficiency depends strongly on induced velocity, torch inclination, and wire mass consumption rate ( Bonthoux, 2016). Here, it is assumed that the fume extraction torch air face velocity is higher than 0.5 m/s and the torch angle is between 0° (perpendicular to the base metal) to 45° to the base metal. This results in capturing efficiency from 80% to 98% (uniform distribution) for torch angles 0° to 45°, respectively, at wire feed rates <1.4 g/s ( Bonthoux, 2016). FET volume flow is assumed to range from 1 to 1.5 m ^3^/min (Presentation “Factors affecting Capture Efficiency of Fume Extraction Torch for GMAW” by Francis Bonthoux, INRS, France).

Based on field measurement studies, movable and fixed LEV system’s capturing efficiency varies from 40% to 50%, according to Flynn & Susi (2012) and from 63% to 88% according to Zaidi *et al.* (2004). The LEV fume capturing efficiency is assumed to vary from 40% to 88% (Uniform distribution). Variation in capturing efficiencies is caused by different welding scenarios where the LEV may not be positioned at an optimal location. LEV flow rate is assumed to range from 5 to 20 m ^3^/min, which is the typical range for welding fume extractors.

***Operational conditions***

Operational conditions specify the minimum requirements for the application of the model results. The results can be applied to any condition favouring a lower exposure level. The CoU assessment decision complies if, for example, the room volume is larger than specified here. Still, the compliance cannot be granted if one of the parameters favour higher exposure. Thus, we selected realistic room parameters so that the CoU result is broadly applicable.

Room volume was selected to range from 30 to 120 m ^3^, which is assumed to be the space a worker needs to conduct welding operations.

Ventilation guidelines for welding workshops were not found or they are based on expected exposure levels. OSHA (2024) sets the minimum rate of 57 m ^3^ per minute per welder, except if local exhaust hoods and booths are applied. According to Microsoft co-pilot, the AWS F3.2M/F3.2:2018 standard recommends a minimum of 20 ACH (not confirmed). Here, we selected a general ventilation rate of 5 to 20 ACH (uniform distribution) without FET or LEV control. Near field volume was selected as a hemisphere with a diameter varying from 0.8 to 1.5 m, covering arm and welding gun length. This results in minimum and maximum volumes of 1 to 7 m ^3^ that were assumed to have a uniform distribution. The NF-FF air exchange was based on Boelter *et al.* (2009) minimum and maximum flow rates of 9.5 to 30.8 m ^3^/min, respectively (uniform distribution).

***Exposure modelling and quantifying CoUs***

Exposure levels were calculated by using TEAS 2Box.CE.Gv model for welding without controls and 2Box.CE.Lev.Gv for welding with FET or LEV ( Ganser & Hewett, 2017). Model parameters are summarized in Table 4. Modelling reports for welding with ER316L electrode and without controls, with FET, and with LEV are available in Extended data, Section 6 ( Koivisto *et al.*, 2025) (results for other electrodes are not shown explicitly).

**Table 4.** Model parameters for CoU assessment. Ranges are assumed to be uniformly distributed. Values are given in range (average).

Parameter, units	Value	Comment
Emission rate, mg/min	Emission ranges are given in Table 3.	Emissions were scaled by multiplying the emission factor by the wire feed rate (50 to 250 mm/s, uniform distribution) and 7.8 g/cm3 wire density.
Arc time, min	58 to 96 (77)	12 to 20% of the 480 min work shift duration.
Room volume, m ^3^	30 to 120 (75)	Space needed by one worker to conduct welding
General ventilation air exchange, 1/h	5 to 20 (12.5)	Does not include ventilation by FET or LEV.
Near-field volume, m ^3^	1 to 7 (4.0)	Hemisphere, radius 0.8 to 1.5 m
NF-FF air exchange, m ^3^/min	9.5 to 30.8 (20.2)	Based on calculated from NF/FF measurements ( Boelter *et al.*, 2009).
FET efficiency, *ε _FET_*	80% to 98% (89%)	Torch angles 0˚ to 45˚, wire feed rates <1.4 g/s, and FET extraction velocity >0.5 m/s
FET volume flow, m ^3^/min	1 to 1.5 (1.25)	Typical FET flow rates
LEV efficiency, *ε _LEV_*	40% to 88% (64%)	Based on field studies
LEV flow rate, m ^3^/min	2.5 to 20 (11.3)	Based on the typical suction capacity of a welding fume extractor.

Calculated GM exposure levels in the NF without controls range from 8.4 to 34 mg/m ^3^ (P95 22 to 88 mg/m ^3^) ( Table 5). Personal exposure measurements show that for GMAW, in the years 1983 to 2016 the GM inhalable exposure level was 3.4 mg/m ^3^ (22% of the measurements were below the limit of quantification (not specified), P25 1.5 mg/m ^3^, and P95 26.4 mg/m ^3^) ( Kendzia *et al.*, 2019). Kendzia *et al.* (2019) did not specify under which operational conditions the measurements were carried out. Thus, the exposure levels contained measurements without and with welding emission controls, and concentrations from background sources. Personal exposure measurements at the upper end (P95 26.4 mg/m ^3^) were like the calculated NF P95 concentration levels without emission controls, which ranged from 22 to 88 mg/m ^3^ ( Table 5). On the other hand, calculated NF concentrations with FET or with LEV ( Table 5) were like the measured personal exposure measurements lower end (22% of the measurements below the limit of quantification and P25 1.5 mg/m ^3^). This shows that the calculated exposure range, with or without controls, is within the exposure range as reported by Kendzia *et al.* (2019).

**Table 5.** Exposure levels (mg/m ^3^) as GM (P95) during welding conditions given in Table 4.

NF/FF	Control	ER316 L	ER316LSi	ER70S-6	ER70S-3	Z 3312 YGW11
NF	None	34 (88)	8.4 (22)	16 (41)	8.6 (22)	9.2 (24)
	FET	3.1 (12)	0.78 (2.9)	1.4 (5.4)	0.81 (3.1)	0.83 (3.1)
	LEV	5 (16)	1.3 (4.1)	2.3 (7.5)	1.3 (4.2)	1.34 (4.3)
FF	None	18 (59)	4.7 (15)	8.6 (28)	4.9 (16)	5.0 (16)
	FET	1.8 (7.5)	0.46 (1.9)	0.82 (3.5)	0.47 (1.9)	0.49 (2.0)
	LEV	2.1 (8.1)	0.53 (2.0)	0.99 (3.7)	0.55 (2.1)	0.56 (2.1)

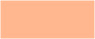
 Non-compliance with the limit value (P95 > 5 mg/m ^3^)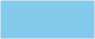 Compliance with ECHA requirement (P90 < 5 mg/m ^3^, here as P95)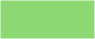 Compliance with EN 689:2019 minimum requirement (GM < 0.1×5 mg/m ^3^)

Depending on the electrode type, P95 exposure during welding without FET or LEV exceeded the OEL of 5 mg/m ^3^ in the NF from 4.4 to 17.6 times and in the FF from 3 to 12 times ( Table 5). Based on ECHA requirement (P90 exposure < OEL), the welding operator in the NF and FF workers should wear a respirator with a nominal protection factor of ≥20 to cover all electrode types if LEV or FET is not used. FET or LEV control did not reduce the P95 exposure levels below 5 mg/m ^3^ for welding by using ER316 L electrode with CO _2_ shielding gas (unlikely combination) and ER70S-6 with 90% Ar + 10% CO _2_ shielding gas. However, P95 exposure in the NF is <5 mg/m ^3^ in welding using ER316LSi, ER70S-3, and Z 3312 YGW11 electrodes when FET or LEV is used ( Table 5). The exposure is adequately controlled for these electrodes, and there is no need for respiratory protection based on the ECHA criteria. Personal exposure measurements should be conducted in 24-month periods for electrodes ER316LSi, ER70S-3, and Z 3312 YGW11 when FET is used (0.1×OEL < GM < 0.25×OEL; ( EN 689:2019). Similarly, the periodic measurement can be assessed for other electrodes using NF concentration ( EN 689:2019). Based on AIHA exposure categorization, welding by using ER316LSi, ER70S-3, and Z 3312 YGW11 electrodes is *controlled* (category 3) *,* and proposed control actions are *chemical specific hazard communication, exposure surveillance, medical surveillance, and work practice evaluation* ( Hewett *et al.*, 2006).

The CoU assessment was conducted by using welding parameters favouring the highest exposure ( Table 3). Thus, the results apply to all process parameters as specified by the investigated studies ( Jilla, 2019; Keane *et al.*, 2014; Satheesh Kumar *et al.*, 2021; Yamazaki *et al.*, 2012).

Reporting CoUs as ranges is not efficient because interpretation is not straightforward. For example, in welding with ER316LSi electrode and FET, the NF P95 and P99 concentrations are 2.9 mg/m ^3^ and 5.0 mg/m ^3^, respectively, i.e., 1% of the scenarios cause overexposure. One approach to exclude overexposure scenarios is to use average CoU values (e.g., arc time 77 min or less). Calculated NF concentrations using average values are 2.7, 3.7, and 2.9 times lower than P95 concentrations without controls, with FET, and with LEV, respectively. This adds sufficient conservatism for reporting the CoUs to avoid potential overexposure scenarios and simplifies reporting (single values).

### CoU refinement

If the CoU assessment is not considered sufficiently broadly applicable to cover different operational conditions, the assessment can be refined based on relevant exposure determinants. Task sensitivity analysis for GMAW welding with an ER316LSi electrode and LEV shows that the most relevant exposure determinants are LEV emission capturing efficiency (-0.552) and emission rate (0.536) (Extended data, Section 7 ( Koivisto *et al.*, 2025), Figure 3). Also, LEV volume flow supplies cleaner air from the FF reducing the concentrations in the NF (-0.18). Here, the simplest refinement is obtained by surveying electrode use amounts in kilograms per work shift because LEV efficiency depends on working practices that are challenging to measure.

**Figure 3.**
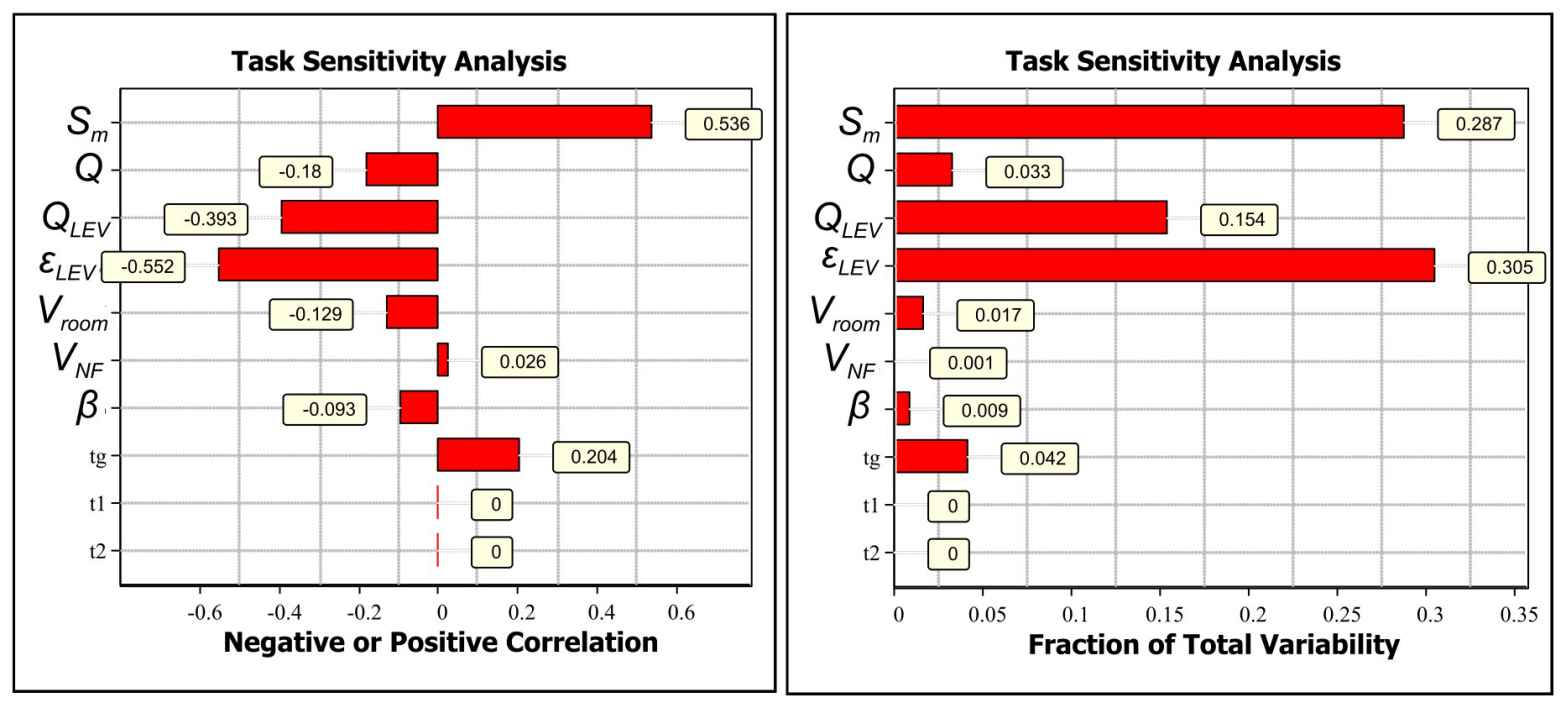
Task sensitivity analysis for NF concentrations for welding with ER316LSi with LEV electrode: **A**) Spearman correlation coefficients showing if the exposure determinant effect on exposure is positive or negative by increasing parameter value and **B**) total variability showing how the exposure determinants cause the variation in the simulated NF concentration level.

### Emission factor limit for adequately controlled exposure (P95 < OEL)

An emission limit can be calculated for specific operational conditions based on exposure limit value. This is demonstrated for Table 4 parameters where the emission factor is back calculated so that the NF P95 exposure is <5 mg/m ^3^. The emission limit was calculated for welding with FET. For emission factor 9.5 mg/g-wire, the emission ranges from 251 to 1256 mg/min (average 754 mg/min) at wire feed rates from 50 to 250 mm/s (average 150 mm/s), respectively, and wire diameter of 1.2 mm. This results in a 4.9 mg/m ^3^ NF concentration as 8-h time weighted average, which complies with the ECHA exposure limit criteria (Extended data, Section 8; Koivisto *et al.*, 2025). This criterion covers, e.g., advanced GMAW welding process with JIS Z3312 YGW11 electrode ( Yamazaki *et al.*, 2012) and GMAW ER70S-6 welding with various shielding gases ( Carpenter *et al.*, 2009; Quecke *et al.*, 2023), and various SMAW, flux core arc welding, and gas tungsten arc welding processes as specified in Quecke *et al.* (2023) and references therein. It is good to remember that the results comply only with total fume emissions, and specific components should be evaluated individually (e.g., hexavalent chromium, gases).

### Limitations and recommendations

Welding emissions impact on exposure level can be predicted with sufficient accuracy for chemical safety decision-making using mass balance and emission factors. Relevant NF exposure determinants for a point source are emission rate and NF-FF air exchange when local ventilation controls are not applied. When LEV is applied, the LEV capturing efficiency is equally relevant to the emission source. The NF-FF air exchange is very rarely measured in different industrial environments. Model accuracy can be refined by measuring and recording the distribution of random air velocities during different welding operations to understand better the variation in the NF-FF air exchange. Both LEV flow rate and capturing efficiency were found to be relevant exposure determinants that are equal to the emission rate. Emission control field performance studies are needed for a realistic picture of their efficiencies under various welding scenarios. Here, model evaluation was carried out without LEV. Model evaluation is recommended with different emission controls, such as FET, LEV, downdraft bench, and welding chamber.

Welding emissions are reasonably well studied and the measurements are relatively well harmonized due to standardization ( Quecke *et al.*, 2023). Here, we presented how to quantify CoU for the total fume emissions of five welding processes. The assessment was conducted for the highest emissions so that the CoU could be applied to all process conditions specified in the studies. A maximum emission factor for total fumes was calculated for the environmental conditions as determined by the authors based on their best knowledge. Generally accepted reasonable worst-case environmental conditions for welding can be used to assess generic CoUs for welding wires and types. Emission labelling for wires can follow, e.g., AIHA exposure categories for which are specified safety actions specific for welding.

Here, the CoU assessment was demonstrated by using total fume emissions. However, in a complete CoU assessment, all relevant emission components should be considered. For example, Cena *et al.* (2014) measured hexavalent chromium emissions up to 0.5% (standard deviation 0.4) from the total fume emissions for welding T304 stainless steel (chromium content 18.0 wt.%) with 308LSi wire (chromium content 20.8 wt.%). The long-term exposure limit value is 5 μg-Cr(VI)/m ^3^ for hexavalent chromium compounds ( ECHA, 2025). Considering hexavalent chromium, the ER316LSi welding scenario would not comply with ECHA requirements ( Table 5).

## Conclusions

Conditions of Use (CoU) are mandatory to assess for materials use and processes in Exposure Scenarios for REACH regulation. A methodology we recently developed to quantify CoUs for industrial processes using emission measurements and exposure modelling ( Koivisto *et al.*, 2021). This method can be used to find the minimum requirements under which the exposure is adequately controlled and to justify the need for periodic exposure measurements. If generic worst-case conditions are specified, it is possible to establish emission labelling for similar processes. This was demonstrated for welding emissions because those are reasonably well-studied.

First, exposure model performance was tested using independent welding emission measurements and reported concentration levels in field exposure assessment studies. Exposure modelling performance was shown to be sufficient for chemical safety decision-making. Then, we demonstrated how to quantify relevant exposure determinants and CoUs for operational conditions for five welding processes. The assessment was performed using the highest emission factors to demonstrate how to maximize the applicability of the results in terms of process parameters and environmental conditions. Different limit values were considered to consider emission and exposure controls, maximize the personal exposure measurement period according to EN 689:2019, and specify the need for safety actions according to AIHA exposure categories. The methods presented in this study apply to all point sources in which emissions are characterized and hazard profiles are known. The method is applicable for regulatory exposure assessment and could provide a more unified and significantly enhanced cost-effective methodology versus the current case-study approach.

## Ethic and consent

Ethical approval and consent were not required

## Institutional review board statement

Not applicable.

## Informed consent statement

Not applicable.

## Data Availability

Zenodo: Extended data for “Quantifying Relevant Exposure Determinants and Conditions of Use for Welding Emissions”. https://doi.org/10.5281/zenodo.14744819 ( Koivisto *et al.*, 2025). The project contains the following underlying data: Extended data.docx: TEAS simulation reports. Data are available under the terms of the Creative Commons Attribution 4.0 International
