# Supplementary material for: Quantifying relevant exposure determinants and conditions of use for welding emissions
Source: Open Res Eur. 2025 Jun 13;5:38. Originally published 2025 Feb 4. [Version 3] doi: 10.12688/openreseurope.19498.3 (PMC12432495; doi:10.12688/openreseurope.19498.3)
Supplement: Peer review history

# Reviewer report: Reviewer response for version 3

**DOI:** 10.21956/openreseurope.22014.r59682

**Published:** 2025-09-23

**Authors:** Vinayak Malik

**Affiliations:** 1 Kls Gogte Institute of Technology, Belagavi, India

Fairly good work. Below are my observations. The manuscript can be improved further based on these suggestions.

 (i) Only total fume emissions were considered, but specific hazardous constituents like hexavalent chromium and manganese are more relevant from a toxicological standpoint. The authors acknowledge this but the limitation needs deeper discussion, particularly the regulatory and health implications of neglecting metal-specific risks. A separate subsection under “Limitations and Recommendations” focused on this would improve clarity.

 (ii) Model performance was tested against two prior field studies (Harris et al. 2005 and Boelter et al. 2009). While the predictability range (0.93 - 3.4) is acceptable for decision-making, the manuscript would benefit from a stronger statistical validation (example, R², RMSE, bias factor). Providing a more quantitative model measurement comparison would help convince readers of robustness.

 (iii) The uncertainty sources are briefly acknowledged (electrode voltage not reported, LEV efficiencies variable, etc). However, the treatment of uncertainty remains qualitative. Including probabilistic confidence intervals in results (example, 95% CI for exposure levels) would make the CoU assessment more reliable and transparent.

 (iv) The paper successfully demonstrates CoU quantification, but the industrial take home message could be stronger. A summarized decision making framework: linking electrode types, ventilation measures and required safety actions, would be very useful for practitioners. Presenting this as a simple decision matrix or flow chart could enhance applicability.

 (v) The findings on Fume Extraction Torch (FET) and LEV are insightful, but the assumptions on capturing efficiency (uniform distributions, ranges from field studies) appear broad. Sensitivity analysis focused specifically on these controls would strengthen the message about their relative importance.

 (vi) The authors note that the method is applicable to other point sources, but this claim is not supported with examples. Adding a short discussion on how this methodology could be adapted to non-welding scenarios (example, nanomaterial handling, chemical spraying) would highlight broader relevance.

 (vii) The paper refers to REACH, AIHA categories and EN 689:2019, but the harmonization of these frameworks is not fully clear. A comparative table showing how CoU results align with each regulatory framework would improve the manuscripts' regulatory utility.

 (viii) The manuscript refers to extended datasets hosted on Zenodo, which is excellent. However, it is unclear if all raw emission data and modeling inputs are reproducible for external users. A clear statement on data completeness and reusability (FAIR principles) should be included.

Is the study design appropriate and does the work have academic merit?

Yes

Is the work clearly and accurately presented and does it cite the current literature?

Yes

If applicable, is the statistical analysis and its interpretation appropriate?

Yes

Are all the source data underlying the results available to ensure full reproducibility?

Partly

Are the conclusions drawn adequately supported by the results?

Partly

Are sufficient details of methods and analysis provided to allow replication by others?

Yes

Reviewer Expertise:

Sustainability and Materials Processing. Additive Manufacturing.

I confirm that I have read this submission and believe that I have an appropriate level of expertise to confirm that it is of an acceptable scientific standard, however I have significant reservations, as outlined above.

# Reviewer report: Reviewer response for version 3

**DOI:** 10.21956/openreseurope.22014.r55499

**Published:** 2025-09-12

**Authors:** Kornél Májlinger

**Affiliations:** 1 Budapest University of Technology and Economics, Budapest, Hungary

Most of my comments I made in R1 were addressed properly. 

 I still recommend improvement on Figures:

 - Figure 2. Axis captions on the diagrams still need clarification e.g. concentration of what is on the  Y axis... X axis designations. 

 I still recommend improvement on captions:

 - Figure and table captions generally need some clarification to see more easily: which data is measured, simulated, own research data or from literature.

Is the study design appropriate and does the work have academic merit?

Partly

Is the work clearly and accurately presented and does it cite the current literature?

Partly

If applicable, is the statistical analysis and its interpretation appropriate?

I cannot comment. A qualified statistician is required.

Are all the source data underlying the results available to ensure full reproducibility?

Partly

Are the conclusions drawn adequately supported by the results?

Partly

Are sufficient details of methods and analysis provided to allow replication by others?

No

Reviewer Expertise:

Welding

I confirm that I have read this submission and believe that I have an appropriate level of expertise to confirm that it is of an acceptable scientific standard.

# Reviewer report: Reviewer response for version 3

**DOI:** 10.21956/openreseurope.22014.r55500

**Published:** 2025-08-29

**Authors:** M. Rahul

**Affiliations:** 1 Indian Institute of Technology Bombay, Mumbai, Maharashtra, India

The authors have well addressed the questions and comments. I recommend to accept the current manuscript for indexing.

Is the study design appropriate and does the work have academic merit?

Yes

Is the work clearly and accurately presented and does it cite the current literature?

Partly

If applicable, is the statistical analysis and its interpretation appropriate?

I cannot comment. A qualified statistician is required.

Are all the source data underlying the results available to ensure full reproducibility?

Partly

Are the conclusions drawn adequately supported by the results?

Yes

Are sufficient details of methods and analysis provided to allow replication by others?

Yes

Reviewer Expertise:

Welding fume emissions, Hexavalent chromium emission, Sustainable Manufacturing

I confirm that I have read this submission and believe that I have an appropriate level of expertise to confirm that it is of an acceptable scientific standard.

# Reviewer report: Reviewer response for version 3

**DOI:** 10.21956/openreseurope.22014.r55624

**Published:** 2025-08-29

**Authors:** Dhanesh G. Mohan

**Affiliations:** 1 University of Sunderland, Sunderland, England, UK

1. The authors should clearly justify why only total fume emissions were modelled, despite the critical role of specific hazardous components like hexavalent chromium.
2. The assumption that the wire feed rate has limited influence on emission factors contradicts field observations in variable shielding environments.
3. The authors should include more detailed validation of model outputs using measured data from additional modern welding setups beyond Harris et al. (2005) and Boelter et al. (2009).
4. The reliance on emission factors from prior literature without standardised calibration introduces uncertainty; this should be quantitatively discussed.
5. Authors should elaborate on the reproducibility and confidence intervals of their exposure modelling predictions.
6. The authors should clarify how the results apply across different welding positions (e.g., overhead, vertical), as these significantly influence fume dispersion.
7. CoU estimation is limited by the assumption of uniformly distributed parameters—authors should test with real-world skewed distributions.
8. The authors should more explicitly define how this model improves over existing IHMod 2.0 implementations, especially in terms of predictive accuracy.
9. There is insufficient consideration of cumulative exposures across multiple tasks or shifts; recommendations are based on isolated single-process scenarios.
10. The authors should mention limitations in generalising their results to automated or robotic GMAW systems.
11. The selection of wire types and shielding gases is narrow; authors should discuss how results vary with other commercial filler wires.
12. The study lacks consideration of intermittent ventilation shutdowns or variations, common in real workplaces.
13. Reporting P95 values without confidence bounds or sample sizes limits the robustness of compliance claims.
14. The model assumes LEV efficiency as a static distribution; real-world LEV performance may degrade over time or due to misalignment.
15. The authors should discuss the role of particle agglomeration and resuspension, which affect exposure beyond initial generation.
16. The high self-citation volume (Koivisto et al. cited extensively) may bias the narrative and should be complemented with independent validations.
17. The proposal for emission labelling is conceptually strong but lacks a standardised framework or pilot implementation guidance.
18. The use of a 5 mg/m³ OEL across the board ignores stricter limits in several jurisdictions (e.g., the Netherlands, Germany)—this should be acknowledged.
19. The authors should clarify why only five electrodes were selected and how they represent broader industry usage.

Is the study design appropriate and does the work have academic merit?

Yes

Is the work clearly and accurately presented and does it cite the current literature?

Partly

If applicable, is the statistical analysis and its interpretation appropriate?

Partly

Are all the source data underlying the results available to ensure full reproducibility?

Yes

Are the conclusions drawn adequately supported by the results?

Partly

Are sufficient details of methods and analysis provided to allow replication by others?

Partly

Reviewer Expertise:

materials science; additive manufacturing; high entropy alloys; hybrid welding; corrosion; coating and composites.

I confirm that I have read this submission and believe that I have an appropriate level of expertise to confirm that it is of an acceptable scientific standard, however I have significant reservations, as outlined above.

# Response

**Published:** 2025-08-29

**Authors:** Antti Joonas Koivisto

Dear Dr. Mohan, we appreciate your valuable and supportive comments. We consider the majority of the comments to be outside the scope of this study or to require additional resources for investigation. However, further investigations are not necessary for this study, which demonstrates how to quantify Conditions of Use (CoU). Please see below the justifications why changes are not essential.  

1. The authors should clearly justify why only total fume emissions were modelled, despite the critical role of specific hazardous components like hexavalent chromium.

  This study demonstrates how to quantify CoU. In the “Limitations and recommendations” section, it is noted that a comprehensive CoU assessment should consider all relevant emission components. Then, an example of hexavalent chromium is provided.  

1. The assumption that the wire feed rate has limited influence on emission factors contradicts field observations in variable shielding environments.

  Wire feed rate directly affects emission (mg/min), but studies show that the emission factor (mg/kg-wire) is not significantly affected, as shown in the referred studies.  

1. The authors should include more detailed validation of model outputs using measured data from additional modern welding setups beyond Harris et al. (2005) and Boelter et al. (2009).

  We agree that validation should be investigated more comprehensively, especially for the welding of coated metals. However, this is another study and requires more resources that are not currently available. More comprehensive validation is provided by Wang et al. (2022) (see Introduction).  

1. The reliance on emission factors from prior literature without standardised calibration introduces uncertainty; this should be quantitatively discussed.

  This is out of the scope of this study. We found one study where different emission measurements (Chang et al. 2004). They summarise that “ *The present results are of comparable magnitude to the EPA emission factors and those reported by an industry group under similar 'average' conditions, typically within a factor of 2. Tests run without shielding gas for SMAW and FCAW produced an order of magnitude greater Cr(VI) emission per unit of electrode consumed.*” Chang et al. 2004. Improving Welding Toxic Metal Emission Estimates in California. Department of Civil & Environmental Engineering University of California, Davis. Available at: https://ww2.arb.ca.gov/sites/default/files/2021-07/report_0.pdf; Accessed 29 Oct 2025  

1. Authors should elaborate on the reproducibility and confidence intervals of their exposure modelling predictions.

  Unfortunately, TEAS provides only exposure distributions and not confidence intervals.  

1. The authors should clarify how the results apply across different welding positions (e.g., overhead, vertical), as these significantly influence fume dispersion.

  The model assumes thoroughly mixed concentrations and does not account for plume drift. It would lead to extremely high number of modellings if the dispersion and location of the welder's face are considered. Such an assessment is currently not feasible within our resources.     

1. CoU estimation is limited by the assumption of uniformly distributed parameters—authors should test with real-world skewed distributions.

  We do not have information for parameters probability density functions.    

1. The authors should more explicitly define how this model improves over existing IHMod 2.0 implementations, especially in terms of predictive accuracy.

  TEAS NF/FF model is the same as IHMod 2.0, and there are no improvements. However, IHMod does not have models for LEV.  

1. There is insufficient consideration of cumulative exposures across multiple tasks or shifts; recommendations are based on isolated single-process scenarios.

  This is why CoUs were calculated for the reasonable worst-case welding scenarios for broad applicability. This does not cover all scenarios, such as welding inside an isolated space. If one of the tasks favours higher exposure, the CoU assessment is not valid, and compliance with an adequately controlled exposure needs to be evaluated separately.    

1. The authors should mention limitations in generalising their results to automated or robotic GMAW systems.

  Most likely, workers are not present at automated welding systems all the time. However, the CoU assessment is applicable only if the operational conditions are met or favour lower exposure.  

1. The selection of wire types and shielding gases is narrow; authors should discuss how results vary with other commercial filler wires.

  This is a demonstration study on quantifying CoUs. Such a study is critical considering safety assessment for various welding processes. However, we don't have the resources to investigate it.    

1. The study lacks consideration of intermittent ventilation shutdowns or variations, common in real workplaces.

  These CoUs apply only for properly operating operational conditions. It is a workplace responsibility to stop the process when the CoUs are not complied with.  

1. Reporting P95 values without confidence bounds or sample sizes limits the robustness of compliance claims.

  We agree, this is a limitation of the study. However, our model does not provide currently confidence intervals for P95 values.  

1. The model assumes LEV efficiency as a static distribution; real-world LEV performance may degrade over time or due to misalignment.

  For LEV, we applied the efficiency range measured in real workplaces. This is generally lower than reported for LEVs. For fume extraction torch (FET), we used a single study where the efficiency was measured under different operational conditions. This does not represent realistic workplace conditions and the efficiency can be under-/overestimated. In the “Limitations and recommendations” section, it is recommended that “ *Model evaluation is recommended with different emission controls, such as FET, LEV, downdraft bench, and welding chamber*.”  

1. The authors should discuss the role of particle agglomeration and resuspension, which affect exposure beyond initial generation.

  If resuspension is a significant source of settled welding emissions, it must be removed by proper cleaning to comply with the CoUs assessed here. Agglomeration affects gravitational settling and is not accounted for in the models used here. Thus, it is not relevant for mass-based risk assessment.  

1. The high self-citation volume (Koivisto et al. cited extensively) may bias the narrative and should be complemented with independent validations.

  This is our first study considering welding emissions. Koivisto et al. (2025) refer to the Underlying Data that is part of this work. Other studies refer to emission libraries in general and the development of quantitative CoU assessment. These studies cannot cause bias.  

1. The proposal for emission labelling is conceptually strong but lacks a standardised framework or pilot implementation guidance.

  We agree. Development of such a framework requires further investigation.  

1. The use of a 5 mg/m³ OEL across the board ignores stricter limits in several jurisdictions (e.g., the Netherlands, Germany)—this should be acknowledged.

  This is acknowledged in the “Exposure limit value” section as “ *The majority of the countries have a 5 mg/m3 limit value as 8-h time weighted average for welding fumes, and the lowest value is 1 mg/m3 (Netherlands) (IARC, 2017).*”  

1. The authors should clarify why only five electrodes were selected and how they represent broader industry usage.

  Five electrodes were considered sufficient for demonstrating CoU assessment. Considering the CoU assessment demonstration, it is not relevant whether they are broadly used in industry.

# Reviewer report: Reviewer response for version 1

**DOI:** 10.21956/openreseurope.21094.r52242

**Published:** 2025-04-10

**Authors:** M. Rahul

**Affiliations:** 1 Indian Institute of Technology Bombay, Mumbai, Maharashtra, India

The manuscript presents a well-structured and methodologically sound study using validated models to assess welding fume emissions, with relevance to occupational exposure control and regulatory compliance. However, some aspects still need clarification or enhancement for full transparency and reproducibility.

**Comments**

1. Clarify Status of Original Work vs. Literature Data: While the paper is categorized as a research article, a significant portion of the data appears to be sourced from previous studies without explicit revaluation. If substantial original data or modeling was performed, this should be more clearly distinguished from cited literature to justify the classification. Otherwise, it may better fit the format of a review with new analysis.
2. Hexavalent Chromium (Cr⁶⁺) Emissions Not Addressed: The manuscript discusses welding fume hazards but does not provide any data on Cr⁶⁺ emissions. Since this is a known carcinogen and occupational hazard, its absence should be explicitly noted, and the potential for measurement or future assessment should be discussed.
3. Lack of Particle Size Distribution Data: Particle size critically affects deposition in the respiratory system and regulatory thresholds. The manuscript would benefit from reporting this data or acknowledging it as a limitation if it was not measured.
4. Missing Measures of Variability: While the extended data provide mean values for emissions, they lack variability indicators (e.g., standard deviation, error margins). Including these would improve scientific rigor and allow better assessment of result reliability.
5. Absence of Chemical Composition Analysis: Welding fume chemical characterization (e.g., elemental composition) is necessary to understand toxicological impacts. If such analysis was not conducted, this should be acknowledged as a limitation, especially when emissions are presented in terms of mass alone.
6. Model Validation with Measured Data: While the model used is well-supported in literature, a direct comparison of modeled exposure against actual measurement (if available) would help validate the study’s specific application and increase credibility.

Is the study design appropriate and does the work have academic merit?

Yes

Is the work clearly and accurately presented and does it cite the current literature?

Partly

If applicable, is the statistical analysis and its interpretation appropriate?

I cannot comment. A qualified statistician is required.

Are all the source data underlying the results available to ensure full reproducibility?

Partly

Are the conclusions drawn adequately supported by the results?

Yes

Are sufficient details of methods and analysis provided to allow replication by others?

Yes

Reviewer Expertise:

Welding fume analysis, Particulate matter emission, Hexavalent chromium analysis, Fume reduction strategies

I confirm that I have read this submission and believe that I have an appropriate level of expertise to confirm that it is of an acceptable scientific standard, however I have significant reservations, as outlined above.

# Response

**Published:** 2025-06-13

**Authors:** Antti Joonas Koivisto

The manuscript presents a well-structured and methodologically sound study using validated models to assess welding fume emissions, with relevance to occupational exposure control and regulatory compliance. However, some aspects still need clarification or enhancement for full transparency and reproducibility. We thank you for the valuable comments and clarifications. We hope the responses below address these comments adequately. 

**Comments**

1. Clarify Status of Original Work vs. Literature Data: While the paper is categorized as a research article, a significant portion of the data appears to be sourced from previous studies without explicit revaluation. If substantial original data or modeling was performed, this should be more clearly distinguished from cited literature to justify the classification. Otherwise, it may better fit the format of a review with new analysis.

This work is based on existing published data, where re-evaluation was made for emission calculations translated from mg/g to mg/min. The work is based on an existing methodology developed by Koivisto et al. (2021) and modelling methods established decades ago (well-mixed and NF/FF model). The novelty of this study is related to a) the discussion on the relevant limit values related to CoU assessment and b) the demonstration of the goodness of mechanistic models in exposure predictions using existing field measurement studies and independently assigned emission factors/rates. This study's relevance is to demonstrate how to assess relevant exposure determinants and CoUs for industrial processes by using a welding process as an example. Despite the fact that CoUs are mandatory to report hazardous emissions in the REACH legislation (ECHA R.14, 2016), the methodology to quantify CoUs was recently developed (Koivisto et al., 2021). Examples are necessary to demonstrate how this approach can be used for regulatory CoU assessment. The value of this study lies in showing how industry can demonstrate safe operational conditions for its processes and materials. Parametrization for broadly applicable CoUs is usually not considered for different limit values, as shown here. This type of assessment cannot be performed with the inhalation exposure models reported in ECHA R.14 (2016), which are not based on physical principles (See Koivisto et al., (2022; 2025), https://doi.org/10.1093/annweh/wxab057 and https://doi.org/10.12688/openreseurope.19510.1). No changes were made.

1. Hexavalent Chromium (Cr⁶⁺) Emissions Not Addressed: The manuscript discusses welding fume hazards but does not provide any data on Cr⁶⁺ emissions. Since this is a known carcinogen and occupational hazard, its absence should be explicitly noted, and the potential for measurement or future assessment should be discussed.

We agree. We added to the Recommendations and limitations section: Here, the CoU assessment was demonstrated by using total fume emissions. However, in a complete CoU assessment, all relevant emission components should be considered. For example, Cena et al. (2014) measured hexavalent chromium emissions up to 0.5% (standard deviation 0.4) from the total fume emissions for welding T304 stainless steel (chromium content 18.0 wt.%) with 308LSi wire (chromium content 20.8 wt.%). The long-term exposure limit value is 5 μg-Cr(VI)/m ^3^ for hexavalent chromium compounds (ECHA, 2025). Considering hexavalent chromium, the ER316LSi welding scenario would not comply with ECHA requirements (Table 5).   

1. Lack of Particle Size Distribution Data: Particle size critically affects deposition in the respiratory system and regulatory thresholds. The manuscript would benefit from reporting this data or acknowledging it as a limitation if it was not measured.

We added particle size information in the Welding emissions section as: The GMAW welding process particle emissions are mainly submicrometric with mass mean aerodynamic diameter ranging from 0.19 to 0.41 µm and geometric standard deviation from 1.3 to 1.8, depending on the welding parameters and shielding gas (Cena et al., 2014; Keane et al., 2009).

1. Missing Measures of Variability: While the extended data provide mean values for emissions, they lack variability indicators (e.g., standard deviation, error margins). Including these would improve scientific rigor and allow better assessment of result reliability.

We agree. In Table 3, only Keane et al. (2014) provides uncertainty/variation margins (6.4±0.5 mg/g) while Sathees Kumar et al., (2021), Jilla (2019), and Yamazaki et al. (2012) provide only average values without uncertainty/variation. For emission rate (mg/min) the variation is mainly associated with the wire feed rate, whose range was selected to represent welding from low to the maximum feed rate as reported in the studies. Variation for Keane et al. (2014) was provided.  

1. Absence of Chemical Composition Analysis: Welding fume chemical characterization (e.g., elemental composition) is necessary to understand toxicological impacts. If such analysis was not conducted, this should be acknowledged as a limitation, especially when emissions are presented in terms of mass alone.

We agree. For a comprehensive CoU assessment, the most relevant components should be investigated separately, and the CoU assessment should be based on both total fume generation and the components possessing the highest risk. This was addressed in the limitations and recommendations section as “The CoU assessment should be extended to all relevant emission components” and in the Abstract conclusions as “Only total fume emissions were considered here, which may not be the limiting factor for risk; volatile and individual metal emissions were not considered.” See also the response to comment 3. No changes were made.  

1. Model Validation with Measured Data: While the model used is well-supported in literature, a direct comparison of modeled exposure against actual measurement (if available) would help validate the study’s specific application and increase credibility.

The model validation for well-mixed and NF/FF models was conducted using published field measurement data and independently measured emissions (Table 1). This was considered a critical step in increasing the credibility of the modelling quality in the CoU assessment. The well-mixed and NF/FF models could adequately predict the measured concentrations for regulatory safety decision-making. However, model validation was conducted without use of LEV or FET. This limitation is addressed in “Limitations and recommendations” -section. No changes were made.   References Cena, L.G., Chisholm, W.P., Keane, M.J., Cumpston, A., Chen, B.T., 2014. Size Distribution and Estimated Respiratory Deposition of Total Chromium, Hexavalent Chromium, Manganese, and Nickel in Gas Metal Arc Welding Fume Aerosols. Aerosol Science and Technology 48, 1254–1263. https://doi.org/10.1080/02786826.2014.980883 ECHA, 2025. CMD - Carcinogens and Mutagens Directive, Annex III - OELVs [WWW Document]. URL https://echa.europa.eu/carcinogens-mutagens-oels/-/legislationlist/substance/100.305.315 (accessed 4.16.25). ECHA, 2018. Committee for Risk Assessment RAC Opinion on scientific evaluation of occupational  exposure limits for Nickel and its compounds (No. ECHA/RAC/ A77-O-0000001412-86-189/F). Keane, M., Stone, S., Chen, B., Slaven, J., Schwegler-Berry, D., Antonini, J., 2009. Hexavalent chromium content in stainless steel welding fumes is dependent on the welding process and shield gas type. J. Environ. Monit. 11, 418–424. https://doi.org/10.1039/B814063D

# Reviewer report: Reviewer response for version 1

**DOI:** 10.21956/openreseurope.21094.r50828

**Published:** 2025-03-25

**Authors:** Kornél Májlinger

**Affiliations:** 1 Budapest University of Technology and Economics, Budapest, Hungary

The paper is listed as research article, but the data presented is mostly from other researchers and their data was in most cases not reevaluated, so in my opinion the paper qualifies more as a review paper. Own work and literature data should be separated more clearly.

 In several cases the welding process used are not mentioned (which has great impact on the emissions) and the chemical compositions of the welding materials and base materials are also not trivial for a common reader.

 The Maximum emission factor should be elaborated, because of the different chemical compositions and particle sizes of the fumes, where the particle size ratios even differ with the welding process used.

 Some data is not cited correctly ( or the authors themselves miscalculated it) e.g. 30 m/min welding speeds are not applicable for manual welding.

 I made my further comments and questions in the manuscript with reviewers comments- https://s3-eu-west-1.amazonaws.com/openreseurope/linked/247291.19498-joonas_koivisto_manuscript_with_rewievers_comments.pdf

Is the study design appropriate and does the work have academic merit?

Partly

Is the work clearly and accurately presented and does it cite the current literature?

Partly

If applicable, is the statistical analysis and its interpretation appropriate?

I cannot comment. A qualified statistician is required.

Are all the source data underlying the results available to ensure full reproducibility?

Partly

Are the conclusions drawn adequately supported by the results?

Partly

Are sufficient details of methods and analysis provided to allow replication by others?

No

Reviewer Expertise:

Welding

I confirm that I have read this submission and believe that I have an appropriate level of expertise to state that I do not consider it to be of an acceptable scientific standard, for reasons outlined above.

# Response

**Published:** 2025-04-08

**Authors:** Antti Joonas Koivisto

We appreciate your valuable comments and expertise in the welding field. We do not have welding expertise. This is not a systematic review study considering welding emissions, but to demonstrate how quantitative CoU assessment should be performed. We acknowledge that numerous factors affect welding emissions, which are not explicitly presented here in the full form. Table 3 provides a list of the major parameters. For a complete list of parameters, the reader should look at the investigated studies. This was left out because the focus is to demonstrate CoU assessment. For simplicity, this study focuses on total fume emissions. It is acknowledged in the main text that a comprehensive assessment should also consider individual emission components. Abstract

- We added “emission”

Introduction

- Sentence reworded to “Personal measurement provides the actual exposition under specific conditions, but the use is limited in generic exposure assessment”
- Citation to Koivisto et al (2021) was removed

Methods

- We agree that base metal contributes to the emissions, but in general, the base metal is much cooler than the electrode tip. This is why the base metal is mentioned as less significant factor. We added another reference to support this statement: Popović, O., Prokić-Cvetković, R., Burzić, M., Lukić, U., Beljić, B., 2014. Fume and gas emission during arc welding: Hazards and recommendation. Renewable and Sustainable Energy Reviews 37, 509–516. https://doi.org/10.1016/j.rser.2014.05.076. We also clarified that the CoU assessment was made for gas metal arc welding (GMAW).
- Figure 2 is generated here based on simulations reported in Extended data, Section 1 (Koivisto et al., 2025). We added to the Figure 2 caption that concentrations are total fume concentrations.
- TWA was written as open (time weighted average)

Methods – Arc time

- Boelter et al. (2009) measured the arc time (no changes were made)
- Welding emission experiments are typically conducted using both extreme and realistic values. Here we used the highest values affecting the emissions to find broadly applicable CoUs. No changes were made.

Methods - Emission controls for GMAW

- This is referring to the fume extraction torch air velocity. We added air face velocity.

Results - Exposure modelling and quantifying CoUs

- We added that ER316 L electrode with CO _2_ shielding gas is unlikely combination.
- Electrode types are likely unknown to the majority of readers. This emphasizes that the results cannot be generalized to all electrode types, because the emissions based on the electrode composition and use can vary significantly.
- We added (Table 3). Other parameters are not listed here because they are not relevant for CoUs' assessment.
- FET refers to the use of a fume extraction torch, which should not impact the welding process. No changes were made.

Results – CoU refinement

- We added GMAW
- Figure 3 is generated from Extended data, Section 7 (Koivisto et al., 2025). No changes were made.
